# An esophageal squamous cell carcinoma classification system that reveals potential targets for therapy

**DOI:** 10.18632/oncotarget.17989

**Published:** 2017-05-18

**Authors:** Teng Xiong, Mengyao Wang, Jing Zhao, Qing Liu, Chao Yang, Wen Luo, Xiangchun Li, Huanming Yang, Karsten Kristiansen, Bhaskar Roy, Yong Zhou

**Affiliations:** ^1^ BGI Education Center, University of Chinese Academy of Sciences, Shenzhen, China; ^2^ BGI-Shenzhen, Shenzhen, China; ^3^ Department of Biology, University of Copenhagen, Copenhagen, Denmark; ^4^ College of Forensic Science, Xi'an Jiaotong University, Key Laboratory of Ministry of Public Health for Forensic Science, Xi'an, China; ^5^ James D. Watson Institute of Genome Sciences, Hangzhou, China

**Keywords:** esophageal squamous cell carcinoma (ESCC), tumor microenvironment, gene set enrichment analysis (GSEA), RNA expression

## Abstract

ESCC (Esophageal squamous cell carcinoma) is a heterogeneous cancer with diverse prognosis. Here, to explore the biological diversity of ESCC, we employed gene expression profiles from 360 ESCC tumors from East Asians to establish a comprehensive molecular classification and characterization of ESCC. Using the specific 185-gene signature generated by unsupervised consensus clustering of gene expression data, we defined four subtypes associated with distinct clinical metrics: tumors with high metastasis associated with EMT (epithelial to mesenchymal transition) and active MAP4K4/JNK signaling pathway; tumors with high chromosomal instability with up regulated MYC targes; well differentiated tumors with less aggressive and moderated tumors. The clinical relevance of these subtypes was stated by significant differences in prognosis. Importantly, 24% of all ESCCs (*n* = 360) were classified into the high metastasis subtype associated with poorly differentiation and unfavorable prognosis. We provided evidence that this subtype relates to tumor microenvironment. Collectively, these results might contribute to more precise personalized therapeutic strategies for each subtype of ESCC patients in the near future.

## INTRODUCTION

Esophageal cancer is caused by the malignancy of cells found in the esophagus. ESCC is the sixth most lethal cancer detected worldwide and approximately 70% of the ESCC occurs in China [1]. Besides, Shanxi Province in north China has the highest incidence rate of ESCCs in the world. In spite of recent advances in diagnosis and treatment methods, the overall five-year survival rate (19%) has not changed significantly [2]. Early detection and treatment is considered to be the recommended strategy wherein patients diagnosed with Stage ESCC without the presence of lymph node or an instance of distant metastasis (T1N0M0) have 90% chances of survival post therapy for five years [3]. However, most ESCCs are diagnosed at advanced stages, and thus the outcome of chemoradiotherapy on these patients is relatively poor and heterogeneous. Therefore, clinical signatures such as TNM Stage and tumor location cannot be considered as a significant prognosis factor, thus a more accurate and an individualized therapy is needed during treatment of ESCC.

Several studies in recent years have applied microarray or RNA-seq technology to explore gene expression profiles in ESCC [4–6] by focusing on differentially expressed genes, miRNAs and non-coding RNAs. But the vast majority of these studies are poor reproducibility, which may be a consequence of distinct molecular signatures that exist in ESCC. The Cancer Genome Atlas (TCGA) has proposed an integrative clustering of ESCC based on multiple molecular platforms [7]. They revealed distinct DNA features of tumors from different populations. The ESCCs from Vietnamese were enriched in *NFE2L2* mutations and *SOX2* amplification and East Asians showed the higher rates of mutations of *NOTCH1*, *ALDH2*, *ADH1B* and *CDK6*. All ESCC patients from USA and Canada had mutations in *SMARCA4*. However, the respective molecular classification of each population and difference in gene expression has not been elucidated.

Recently, three subtypes were identified based on the mRNA expression data from 59 ESCC individuals in Malawi, of which classification could be distinguished by their expression of cell cycle and neutral transcripts [8]. Besides they also observed related genomic alterations in the specific subtypes, in addition to the previous studies, Yang et al., re-analyzed previously published mRNA and lncRNA data from 119 ESCC patients in China, and identified two subtypes with significantly different prognosis, and also demonstrated key nodes on mRNA-lncRNA networks in subtype-specific ESCC [9]. Both of these two studies provided valuable insights into ESCC.

Here, we explored 360 ESCC tumors from East Asians to establish a robust molecular classification based on unsupervised consensus clustering of mRNA expression profiles. Subsequently, the association with every subtype based on the clinical data, pathological data, chromosomal alterations and tumor microenvironment were assessed. The tumor microenvironment has been demonstrated to be associated with various tumor gene signatures and useful for prognosis across many cancers [10, 11].

## RESULTS

### Identification of four subtypes in ESCC

**Figure 1:**
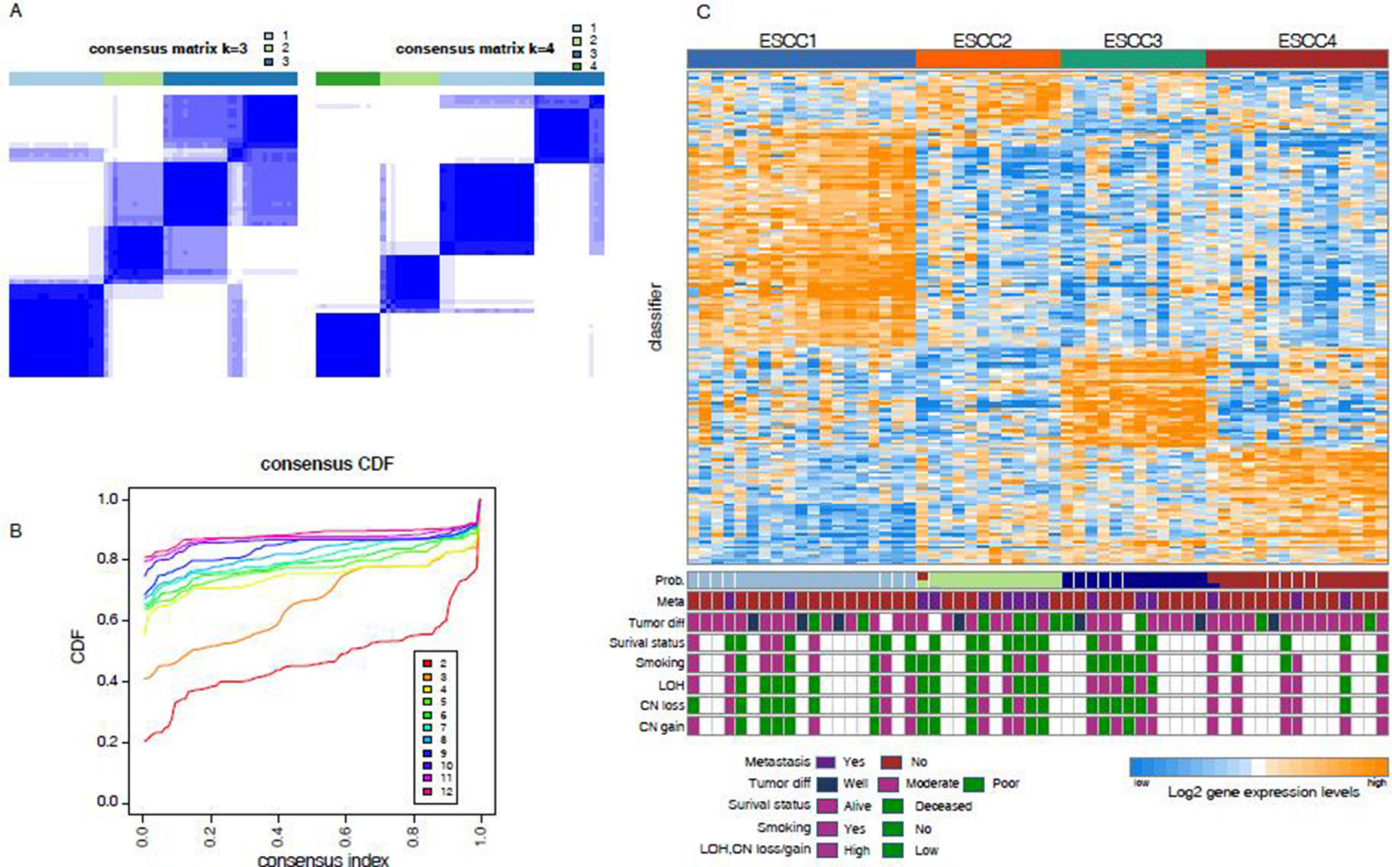
Unsupervised classification identified four subtypes (**A**) Consensus clustering matrix shows the optimal four clusters. (**B**) The Item-consensus plot shows the relationship between each cluster. (**C**) Up heatmap shows the four subtypes according to the PAM classifier. Bottom barplots show the clinical information associated with each sample.

To explore the heterogeneity of ESCC, we used previously developed consensus unsupervised clustering technique (Supplementary Figure 3) [12] to cluster two published expression data sets GSE38129 (*n* = 30) and GSE45670 (*n* = 28). These datasets were corrected for technical batch effects and merged into a dataset of 58 cases using DWD method before clustering. The analysis defined four clusters with most robust classification (Figure 1A, 1B). The consensus matrix showed the presence of an overlap between cluster3 and cluster4. Examination of the item-consensus plot showed that ESCC1 was overlapped with ESCC3 during consensus classification, and it also revealed that ESCC2 was the most distinct subtype in comparison to other subtypes (Supplementary Figure 1A). We used silhouette width to select the most representative samples for each cluster, of which 53 samples with positive silhouette width were retained (Supplementary Figure 1E). In order to build a classifier, differentially expressed genes across four clusters were identified using the significance analysis of microarrays (SAM, false discovery rate (FDR) < 0.01), followed by prediction analysis for microarrays (PAM) to train the most representative and predictive genes with AUC > 0.9. Finally, 185 gene signature classifier that reliably divided 58 cases into four groups: ESCC1 (*n* = 19, 33%), ESCC2 (*n* = 11, 19%), ESCC3 (*n* = 13, 22%), ESCC4 (*n* = 15, 26%) (Figure 1C, Supplementary Table 2) with prediction error less than 0.02 was developed.

### Validation of subtypes across different datasets

In this study, we have applied the 185 gene signature classifiers into four independent gene expression datasets for validation of the subtypes. All the 185 genes were projected onto each data set. Following which the R package PAMR was used to calculate the posterior probability of each sample associated with four subtypes. A sample is categorized into one subtype with the maximal posterior probability that at least greater than 0.5. The classifier was validated in GSE23400, GSE47404 and GSE53624 datasets and found that all four subtypes were assigned with comparable proportions of samples (Supplementary Figure 2A–2D). Moreover, additional datasets GSE33426 containing samples from both micro-dissected tumors were used. Although, all samples of these datasets were represented in three of our four subtypes, only two samples were classified into ESCC3 (Supplementary Figure 2C). This result suggested that possible intra-tumor heterogeneity dominated by cancer cells with characteristics of a particular subtype, but most subtypes were still routinely identified. This has been suggested in breast cancer earlier as well [13].

### Clinical and molecular relevance of ESCC subtypes

**Figure 2:**
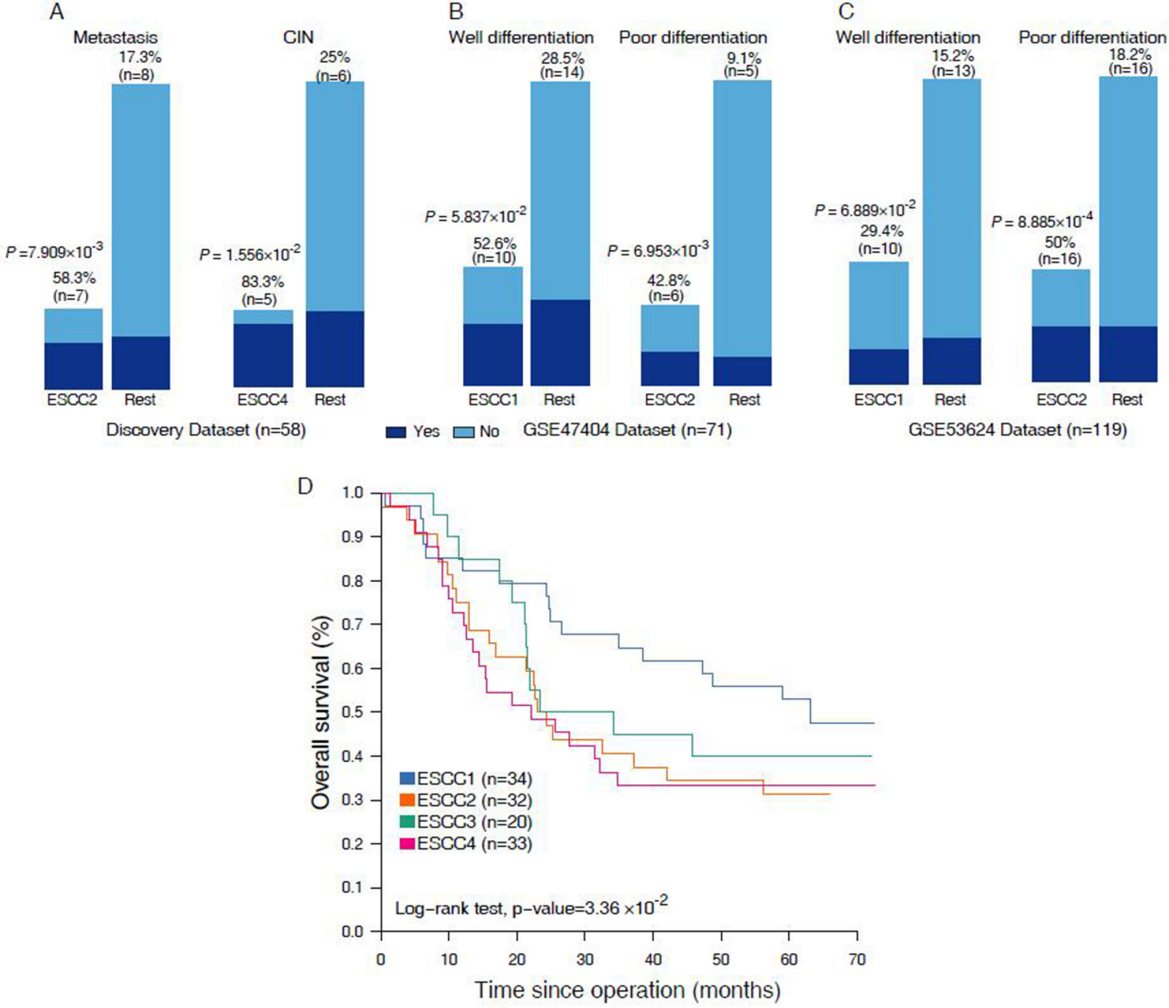
(**A**–**C**) Barpolts show the comparation on the clinical features in discover set and validation sets. (**D**) Kaplan-Meier graphs depicting disease-free survival (DFS) within GSE53624 (*n* = 119) stratified by the classification.

To further characterize these four subtypes, we determined the clinical and histopathological features like metastasis, tumor differentiation, smoking, loss of heterozygosity (LOH) and copy number (CN) gain or loss (Figure 1C, Supplementary Table 3). Samples of ESCC2 were more frequently metastasized to other parts of the body (58.3% [*n* = 7] vs. 17.3% [*n* = 8]; *P* = 7.909 × 10^−3^, Fisher exact test, Figure 2A) and entirely deceased after neo-adjuvant chemoradiotherapy, indicating that this subtype has very high potential to metastasize of all the ESCC tumor subtypes and confirms that tumor metastasis is a common cause of ESCC mortality [14]. The ESCC4 group was indeed associated with genomic instability, wherein high frequency genomic instability measures (LOH, CN loss, CN gain ≥ 10%) were often observed in this subtype (83.3% [*n* = 5] vs. 25% [*n* = 6]; *P* = 1.556 × 10^−2^, Fisher exact test, Figure 2A). The CNA microarray analysis identified frequent DNA copy alteration including CN loss on 3p (33%), and CN gain on 3q (48%). About 70% of the LOH was found to be CNLOH, and has been reported to be highly associated with tumor development [15]. Patients classified under ESCC1 and ESCC4 subtypes were more frequently found to be smoking (60%, 50%, respectively, versus < 20% in other groups) and also 75% of these samples were stage III tumors and thereby suggesting that there is no association between clusters and tumor stage. Moreover, we found that cancer cell differentiation may be associated with ESCC1 and ESCC2 in validation sets GSE47404 and GSE53624. Observations have also revealed that, in GSE47404, 52.6% (*n* = 10) of ESCC1 samples were well differentiated with borderline significance (*P* = 5.837 × 10^−2^, Fisher exact test, Figure 2B), whereas 42.8% (*n* = 6) of the ESCC2 samples were poorly differentiated (*P* = 6.953 × 10^−3^, Fisher exact test, Figure 2B). And in GSE53624, 29.4% (*n* = 10) of ESCC1 samples were well differentiated (*P* = 6.889 × 10^−2^, Fisher exact test, Figure 2C), whereas 50% (*n* = 32) of ESCC2 samples were poorly differentiated (*P* = 8.8858 × 10^−4^, Fisher exact test, Figure 2C). These results suggested that ESCC1 tumors have a low malignancy potential in comparison to the ESCC2 tumors which are highly aggressive and tend to grow and spread more quickly, which is in agreement with ESCC2 being metastasis-associated with discovery dataset.

Moreover, we performed Kaplan-Meier survival analysis to investigate the prognostic value of the four subtypes. The prognosis of each subtype in discovery set (*n* = 58) is not significant due to insufficient survival information. Nevertheless, we found significant differences in overall survival of the four subtypes in validation set GSE53624 (*n* = 119, Figure 2D), and confirmed a poor prognosis of patients with ESCC2 and ESCC4 tumors. These results are consistent with our classification that well-differentiated subtypes (ESCC1) have better survival than those of metastasis and poor-differentiated (ESCC2). Cumulatively, our classification system might provide useful information for risk stratification and treatment.

### Signaling pathways associated with ESCC Subtypes

**Figure 3:**
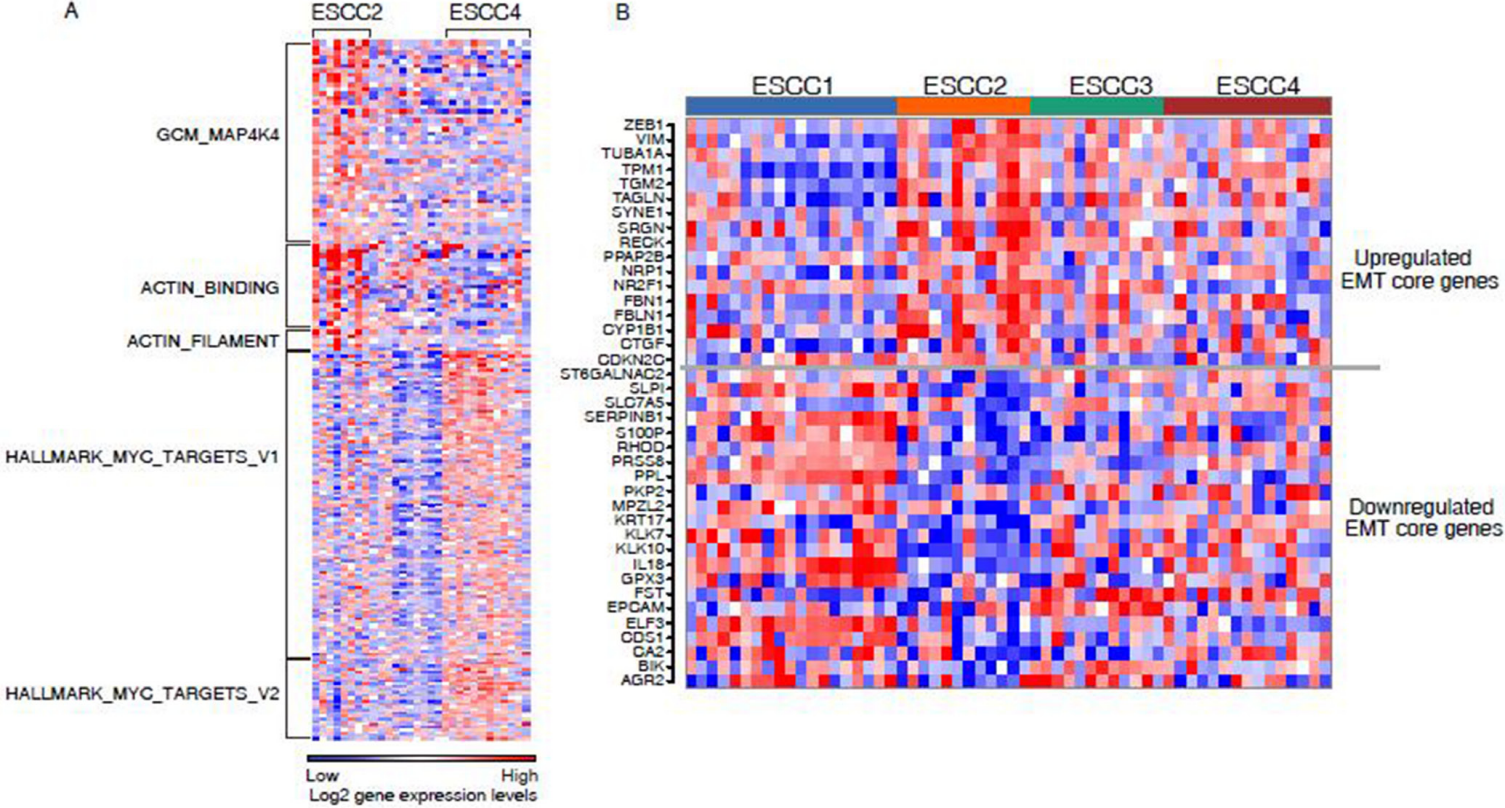
(**A**) Gene set enrichment analysis for ESCC2 and ESCC4. Heatmap shows ESCC2 and ESCC4 enriched on the selected gene sets. (**B**) Heatmaps showing the core gene sets for EMT was significantly dysregulated in ESCC2 subtype.

To investigate the biological properties that were associated with ESCC subtypes, gene set enrichment analysis (GSEA) was applied to determine gene sets which were more abundant in specific subtypes. During the investigation, our focus was on ESCC2 and ESCC4 subtypes associated with clinical signatures. The ESCC2 subtype was significantly enriched in gene sets namely, GCM_MAP4K4, ACTIN_BINDING and ACTIN_FILAMENT (Figure 3A, Supplementary Table 4). Of these, *MAP4K4* encodes a protein that is member of the mammalian serine/threonine protein kinase family. Previous studies have suggested that this gene was necessary for the migration of different cancer cells in various tumors such as hepatocellular, bladder and ovarian carcinoma [16]. The influence of *MAP4K4* on tumor proliferation, migration and invasion was associated with the activation of the c-jun N-terminal kinase (JNK) pathway [17]. Further, Knockdown of *MAP4K4* may also help in treating ESCC2 tumors. Likewise, actin is an important protein in mammalian cells, which can promote cells to move, polarise, divide and maintain organization. Actin-binding and actin filament proteins can reorganize the actin cytoskeleton, and drive cancer cell migration and invasion [18]. Components of the actin system may serve as significant potential targets for this subtype. In order to investigate the association between ESCC2 subtype and epithelial-mesenchymal transition (EMT), we used a 130 EMT-core regulated gene list from a pan-cancer study [19] as gene set. Of which 89 genes were identified from the list that were expressed in our discovery set (Supplementary Table 5). As a consequence, 17 genes of the EMT-core upregulated genes were significantly upregulated in ESCC2 subtype, including well-known EMT makers such as *ZEB1* and *VIM* (Figure 3B), while 22 genes of the EMT-core downregulated genes were significantly downregulated in ESCC2 subtype, including reported downregulated epithelial cell makers such as *EPCAM*, *KRT17*, *PKP2* and *PPL* and some tumor suppressors such as *KLK10* and *SERPINB1* (Figure 3B). These results indicated ESCC2 subtype is associated with EMT. The results obtained during the analyses have confirmed that ESCC2 tumors metastasize more frequently than other subtypes and also upregulate genes driving tumor cells towards metastasis.

The ESCC4 subtype showed more abundant expression of genes involved in hallmark *MYC* targets V1 and V2 (Figure 3A, Supplementary Table 4). *MYC* has been implicated as a driver gene in ESCC and it plays a crucial role in cell cycle progression, apoptosis and cellular transformation by deregulating hundreds of direct target genes [20, 21]. *MYC* mediates genomic instability by promoting chromosome tetraploidy and aneuploidy. Leading edge analysis was performed to select overlapping genes from the two gene sets which were of interest. ESCC4 tumors were enriched in cell markers *CDK4*, *MCM4*, *DDX18*, *PHB*, *PA2G4*, *HSPD1* and *HSPE1*. This result represented the well characterized group of chromosomal instability (CIN) tumors for ESCC4 subtype. Based on the clinical features and signaling pathway proposed above, the four subtypes were identified as: ESCC1, “well-differentiated”; ESCC2, “metastasis-associated”; ESCC3, “moderated”; ESCC4, “CIN+”.

### Tumor microenvironment of ESCC subtypes

**Figure 4:**
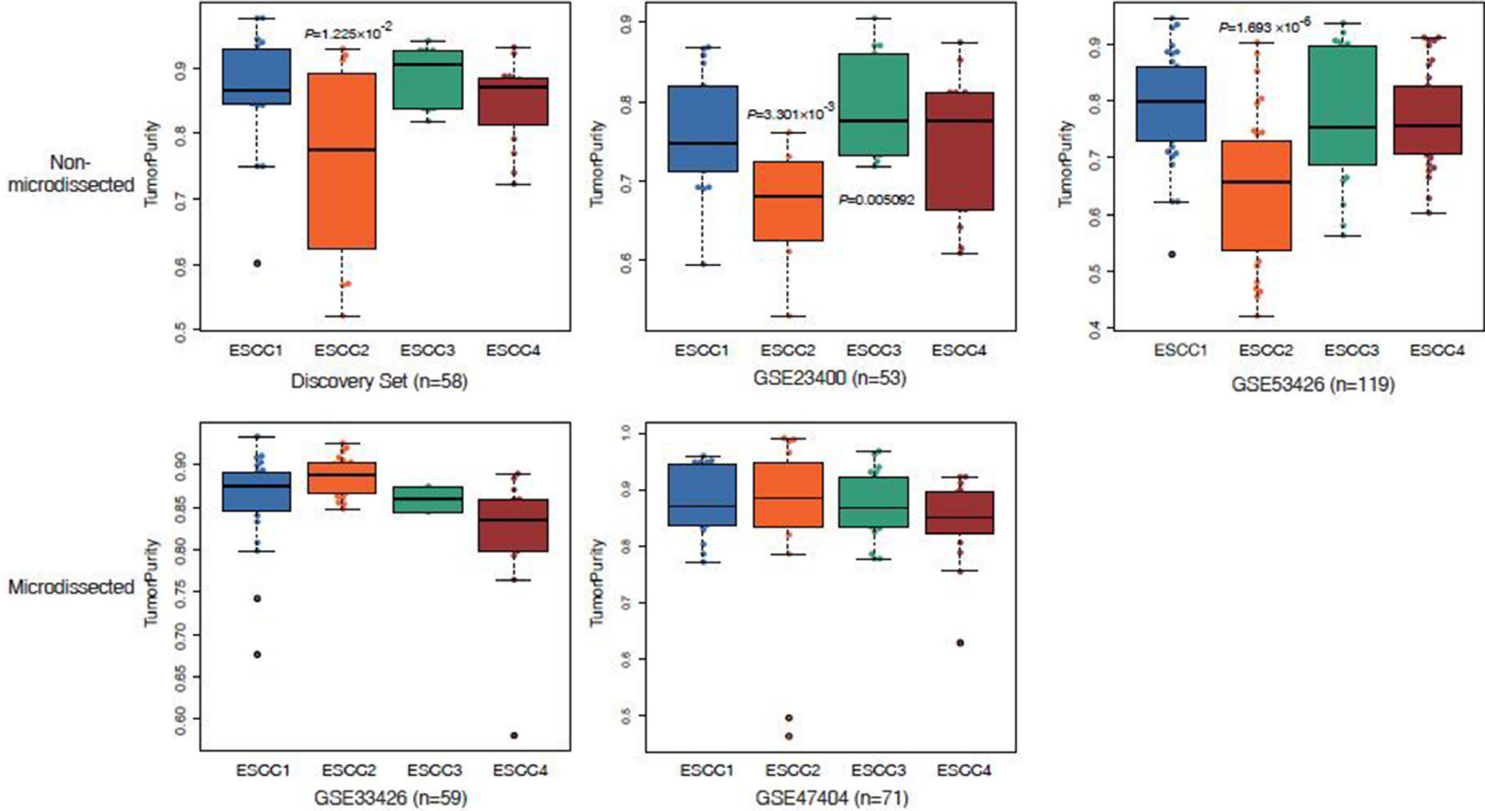
Box plots display reduced tumor purity in ESCC2 tumors

**Figure 5:**
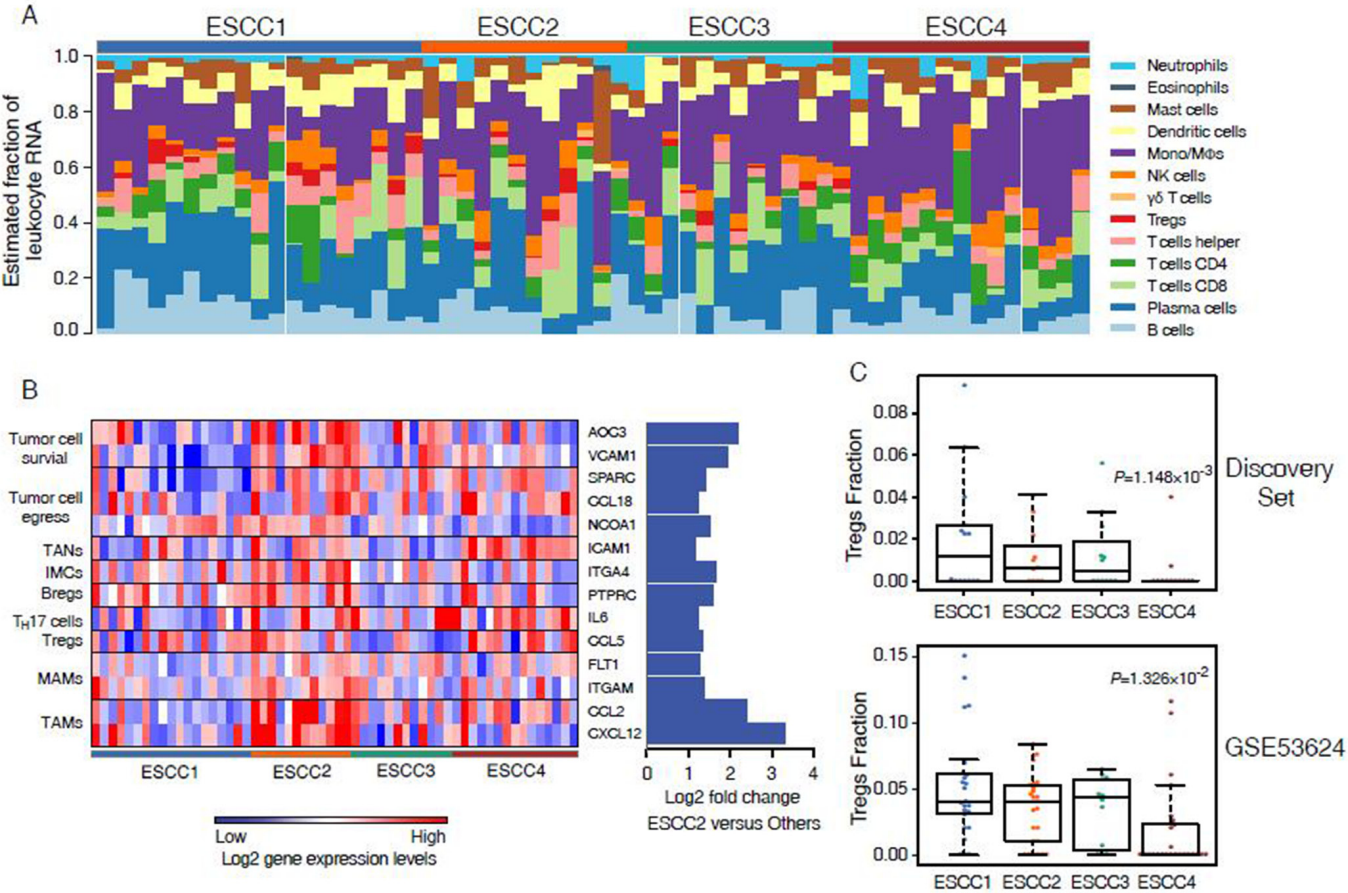
Immune cell composition inferred from ESCC microarray profiles (**A**) Evaluated mRNA fraction of 22 leukocytes across 58 ESCC tumors. (**B**) Heatmap shows the 14 up-regulated genes associated with metastasis in ESCC2. (**C**) Comparison of immune cell fraction of Tregs across 4 subtypes in discovery set and GSE53624.

Intratumor heterogeneity is associated with the tumor microenvironment which comprising a variety of tumor-associated stromas and leukocytes. To explore the performances of the microenvironment in different ESCC subtypes, ESTIMATE [10] was applied to infer tumor purity and stroma or immune cell fraction for each sample in discovery set and validation sets. We found that the average tumor purity of ESCC2 tumors was significantly lower than tumors from other subtypes in discovery set and non-microdissected validation sets (GSE23400 and GSE53624, Figure 4). This indicated that infiltrating stroma and immune cells may contribute to ESCC2 subtype (metastasis, EMT, poorly differentiation), which is consistent with previous studies in colorectal cancer [22]. However, we could identify ESCC2 group in both microdissected datasets (GSE33426 and GSE47404, Figure 4), suggesting that ESCC2 signature genes might not be expressed by stroma cells and immune cells. Moreover, we observed that immune cells were retained in the microdissected dataset in each subtype which reflected the infiltrating immune cells intermix in tumors (Supplementary Figure 4). Then, we utilized CIBERSORT (a machine learning approach) [23] to identify diverse immune cell fractions. We used expression profiles of discovery set (*n* = 58) and GSE53624 (*n* = 119) that are in non-log space respectively, as input to evaluate 22 distinct immune cell types based on 547 signature genes. It was observed that all the ESCCs were commonly found to contain plasma cells and macrophages. (Figure 4A, Supplementary Figure 5A, Supplementary Table 6). We evaluated lower fractions of the regulatory T cells (Tregs) in ESCC4 tumors in both discovery set and GSE53624 (Figure 5C). Tregs is generally shown to facilitate immune escape by suppressing activity of effector T cells. Future studies are needed to illuminate the reason for lack of Tregs in ESCC4 tumors. Significant differences in relative frequencies of some immune cell composition across four subtypes could only be observed in discovery set (Supplementary Figure 6B). This may be due to some samples being pretreated in discovery set.

To investigate the association of leukocytes with ESCC2 subtype, SAM was used to detect differentially expressed genes in ESCC2 tumors. This study especially focuses focused on genes related to metastasis and discovered that 20 up-regulated genes associated with immune cells in discovery set and GSE53624 which could promote every steps of the metastatic cascade (Figure 4B, Supplementary Figure 5B and Supplementary Figure 6A) [24]. Chemokines and cytokines including *CCL2*, *CXCL12*, *CSF1*, *CCL5*, *CCL22*, *IL6* and *TGFB3* are secreted by primary tumor cells to recruit immune cells to escape from anti-tumor immune responses [25–27]. Regulatory B cells (Bregs) which express *PTPRC* may also promote metastasis through immune suppression [28]. Moreover, recent studies have indicated that TAMs and tumor-associated neutrophils (TANs) can also contribute to tumor cell egress and survival via *NCOA1*, *CCL18*, *VCAM1*, *ICAM1* etc [29–31]. In addition, immature myeloid cells are the major component of pre-metastatic niche and metastatic-associated macrophages (MAMs) which interacts with the emigrated cancer cells to facilitate persistent growth of metastatic. These results may contribute to immunotherapy for metastatic ESCCs by targeting these immune cells.

## DISCUSSION

In summary, we have built a molecular classifier for ESCC based on analysis of gene expression profiles, and identified four distinct subtypes (ESCC1, ESCC2, ESCC3, and ESCC4) that are associated with different clinical and molecular characteristics. These four distinct subtypes were validated in four primary data sets, even in microdissected tumors. The ESCC2 tumors were mostly metastatic that are associated with poor differentiation, EMT and poor prognosis. This subtype has been previously reported on colon cancer [22], our analyses suggested that this subtype is also existed in ESCC. Furthermore, the ESCC4 subtype is associated with CIN, comparable poor prognosis and revealed overexpression of *MYC* target genes in the subset. Most of the ESCC4 tumors were also identified with high frequency of loss of heterozygosity (LOH). In particular observations revealed that, ESCC1 tumors were mostly well differentiated compare to ESCC2 tumors in the validation sets GSE47404 and GSE53624 and have better survival than other subtypes. Notably, the ESCC3 subtype is much less well characterized and needs further investigation. Only two of 59 tumors were classified in ESCC3 (GSE33426), which might due to tumor microenvironmental contaminations. Nevertheless, GSE47404 has similar percentage of ESCC3, suggesting that there might be other factors leading to the imbalanced percentage of ESCC3 in GSE33426 instead of microenvironmental contaminations. Relatively small sample sizes were used in our analysis (less than 100) and inter-patient tumor heterogeneity is large, which is more likely to be the cause.

In this analyses showed that ESCC2 samples have a significantly higher stoma and immune content. This is consistent with previous study in colorectal cancer [22] and ovarian carcinoma [32] that high stromal or immune scores reflect the the presence of EMT subtypes. We have also identified that ESCC2 signature genes are not expressed by stroma cells and leukocytes. This abundant stroma and immune cells may be considered a feature of ESCC2 subtype. Further study in ESCC PDXs and cell lines may be needed to more quantitatively investigate the extent of tumor microenvironmental contribution to this subtype. By applying CIBERSORT, we observed relationships between ESCC subtypes and immune cell signatures. In depth observations have shown that, ESCC4 subtype correlates with the absence of Tregs. Also, the ESCC2 subset was influenced by various cell types which were regulated by distinct chemoattractants, especially TAMs acting in every step of the metastatic cascade. Current therapies are limited to targeting only macrophages and hence a more detailed study of interactions between each immune cell and associated with ESCC2 is needed to devise a more precise immunotherapy for metastasis.

Our analysis was limited by lack of ESCC microarray data, clinical tracking information and molecular characteristics. Further study with large ESCC cohorts is needed to confirm the significance and robustness of the classifier. On the hindsight, it would motivate investigations into associations between clinicopathological signatures and these subtypes [33]. Recognition of these classifications clearly reflects the intra-heterogeneity of ESCC and provides a basis for detecting potential biomarkers or therapeutic approaches for specific subtypes in preclinical trials which would finally contribute to personalized treatment [34].

## MATERIALS AND METHODS

### Sample collection

In this study, six independent ESCC microarray datasets from GEO Datasets comprising a total of 360 unique samples with stage I–III primary ESCC from East Asians were used. The discovery dataset contains 58 cases with stag II to III ESCCs from two datasets, GSE38129 (*n* = 30) [6] and GSE45670 (*n* = 28) [35], whereas the validation datasets include GSE23400 (*n* = 53) [36], GSE33426 (*n* = 59) [37] and GSE47404 (*n* = 71) [38] and GSE53624 (*n* = 119) [39]. Detail information about each dataset was illustrated in Supplementary Table 1.

### Gene expression analysis and data processing

Initially, the CEL files from GEO datasets were downloaded and the two datasets (GSE38129 and GSE45670) were normalized using fRMA [40] independently. Nevertheless, Barcode algorithm [41] was also employed to distinguish between expressed or unexpressed genes. Subsequently, genes expressed in at least one sample of the two datasets were retained. Also, the probe sets were selected with MAD greater than 0.5 and the median centered. Later, the two datasets were merged using Java-based distance-weighted discrimination method [42]. Finally, the rows were median centered and 3118 probe sets were retained with MAD > 0.5 (Supplementary Figure 3).

### Consensus cluster and generation of classifier

Consensus clustering [43] was implemented in the R package ConsensusClusterPlus, with 1000 iteration and 0.98 subsampling ration to determine a robust clustering. A significant increase in clustering stability was observed from k = 2–4, but not for k > 4 (Figure. 1B). Cluster robustness analysis was performed using the gap statistic [44] for top 3000 differential expressed probe sets, and a peak was consistently found at k = 4 (Supplementary Figure 1C). We collapsed the expression profiles from the probe sets to unique genes using collapseRows (R package WGCNA) [45]. The probe sets were selected on the basis of the highest mean expression of each gene. Also, the most representative genes were identified using SAM (R package siggenes) [46] with FDR < 0.01 and 206 genes were retained with AUC > 0.9 (R package ROCR). Finally, PAM [47] was used to determine 185 subtype-specific signature genes.

### Validation in additional data sets

Initially, fRMA was used to process GSE23400 and GSE33426 data set. In case of GSE47404 and GSE53624 dataset (Agilent Microarry), quantile normalized microarray data was directly downloaded from GEO Datasets. For each preprocessed data set obtained, expression profiles from probe sets were collapsed to unique genes using collapseRows. The signature genes that were not included in the validation data sets were replaced by the most correlating gene which was expressed in validation sets. Finally, the PAM classifier was used for each preprocessed data sets for classification of gene expression data.

### Gene Set Enrichment Analysis (GSEA)

GSEA [48] was performed using Java GSEA Desktop Application. Molecular Signatures Database (MSigDB) was used as gene set for analysis and further *P* values were estimated by 1,000 permutations. Also, un-filtered GSE38129 data set was used for analysis.

### Estimation of tumor purity, stroma and immune cell mixture

The proportion of stromal and infiltrating immune cells were measured with ESTIMATE [10], a gene expression signature-based method that estimate tumor purity from the gene expression data.

### Inferring immune cells composition

The data subsets GSE38129, GSE45670 and GSE53624 were evaluated by applying CIBERSORT [49] with the LM22 gene signature to identify 22 immune cell types. For this analysis, microarray probes were replaced with HUGO gene symbols. Genes with multiple probe were collapsed to the one with the highest mean expression. Expression profiles were normalized using fRMA and then by antilog of 2^x^. Analyses were done with 100 permutations with default parameters and results were filtered by a maximum *p-value* of 0.05.

## SUPPLEMENTARY MATERIALS FIGURES AND TABLES












