# Supplementary material for: An esophageal squamous cell carcinoma classification system that reveals potential targets for therapy
Source: Oncotarget. 2017 May 18;8(30):49851–60. doi: 10.18632/oncotarget.17989 (PMC5564812; doi:10.18632/oncotarget.17989)
Supplement: Supplementary file 1 [file oncotarget-08-49851-s001.pdf]

An esophageal squamous cell carcinoma classification system that reveals potential targets for therapy

Supplementary Materials

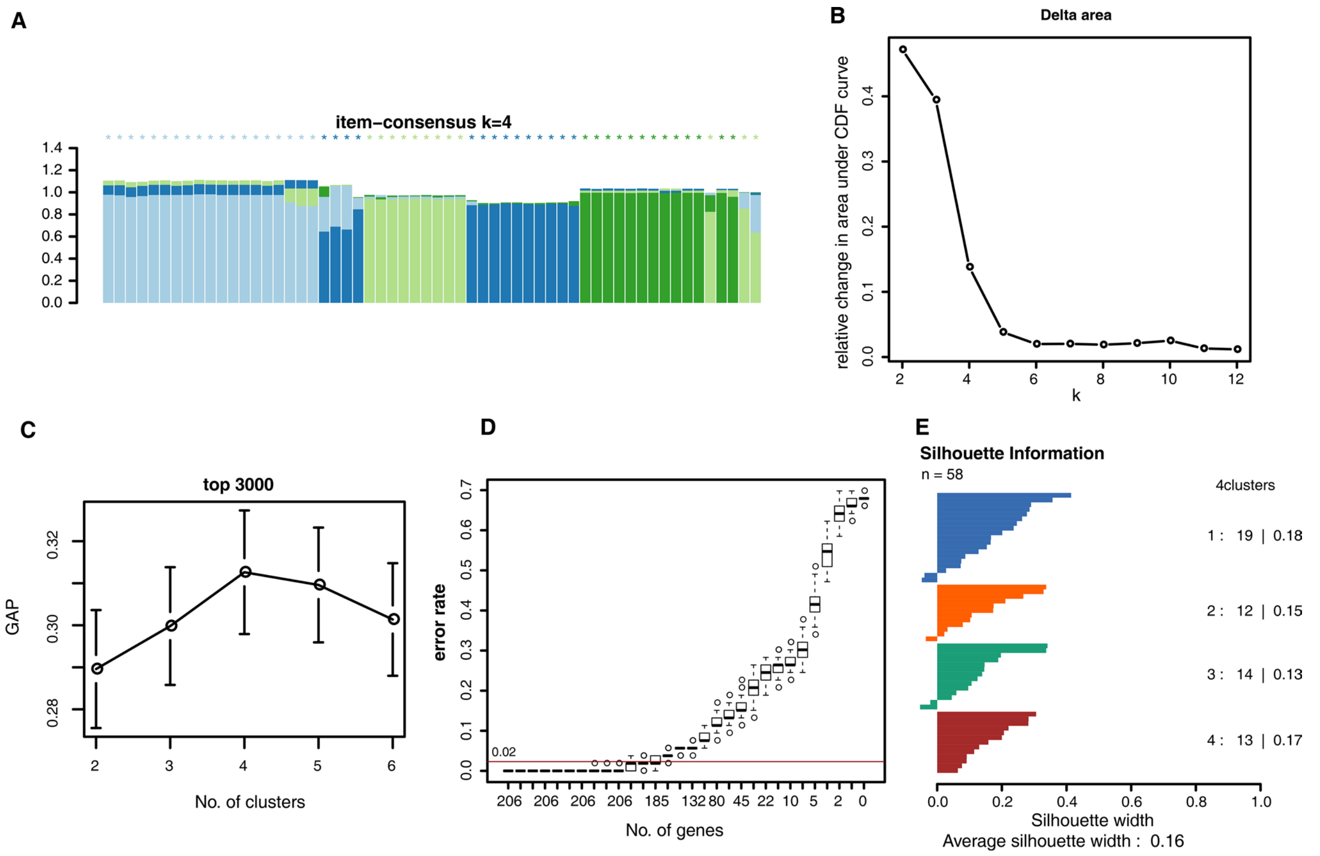

Supplementary Figure 1: Consensus clustering. (**A**) CDF plot shows k = 4 approaches the robust consensus distribution. (**B**) The Delta Area plot shows the change in the area under the CDF curves. (**C**) Gap statistic for 3000 probesets. (**D**) Boxplot shows the crossvalidation error rate, we selected the least number of genes with error rate < 2%. (**E**) Barplot shows the silhouette width for samples in each cluster.

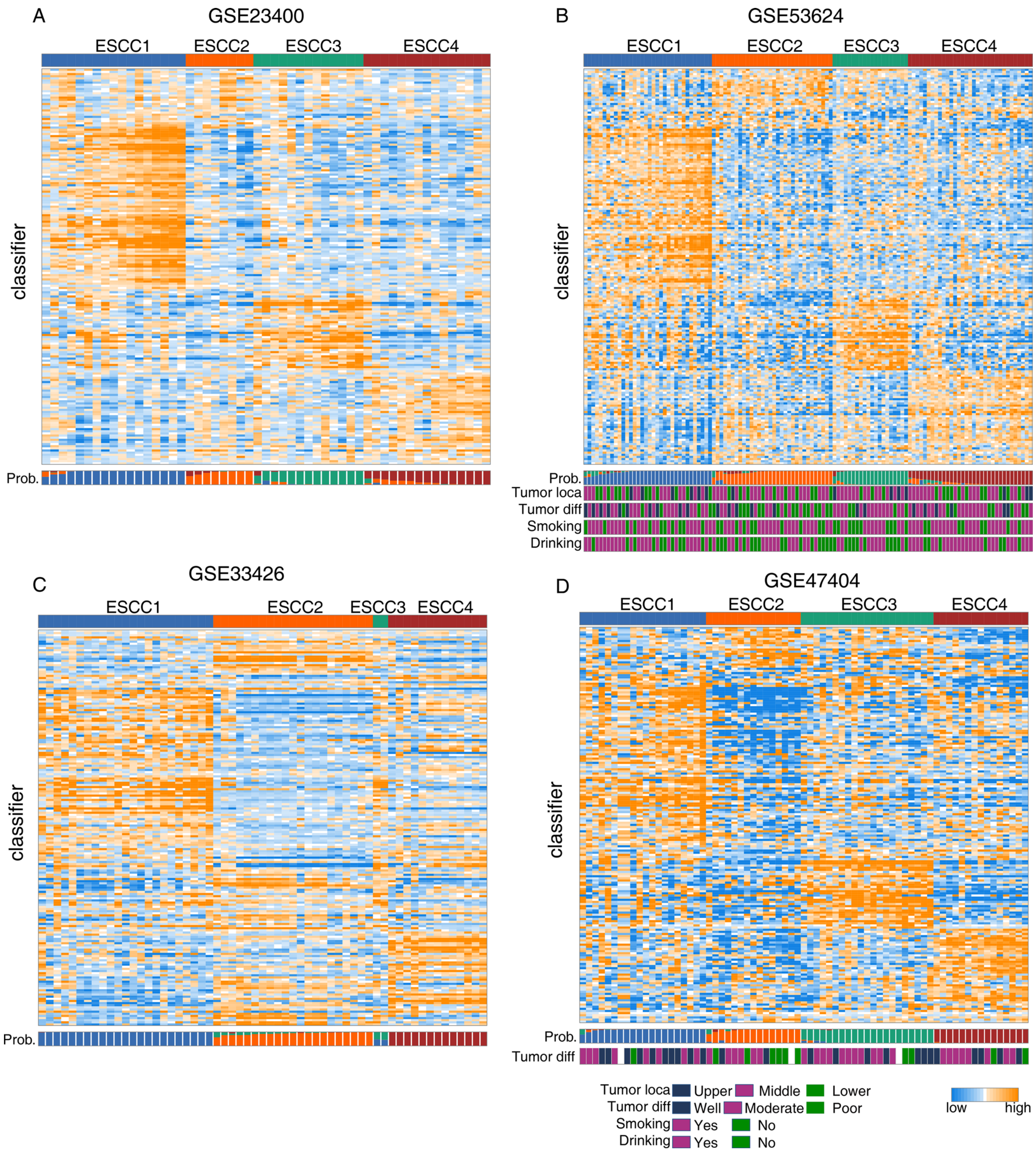

Supplementary Figure 2: Classification of validation data sets based on the 185-gene classifier. (**A**) GSE23400. (**B**) GSE53624. (**C**) GSE33426. (**D**) GSE47404.

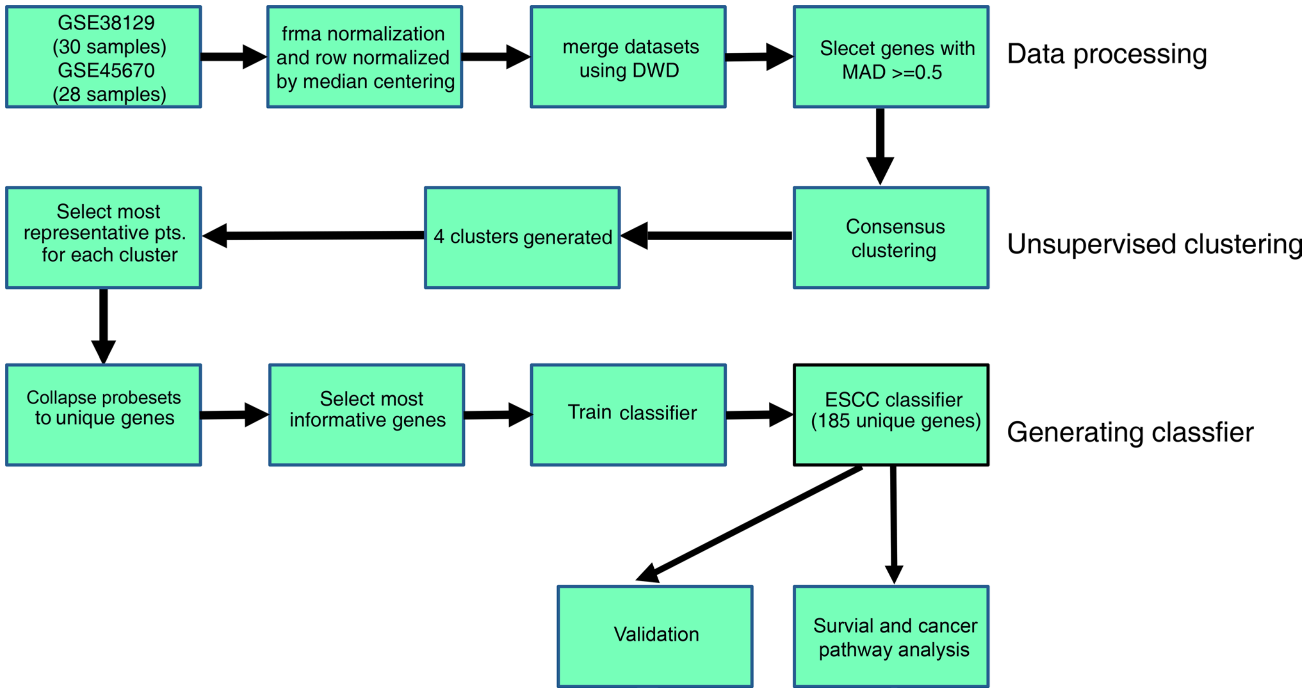

Supplementary Figure 3: Summary of the analysis pipeline.

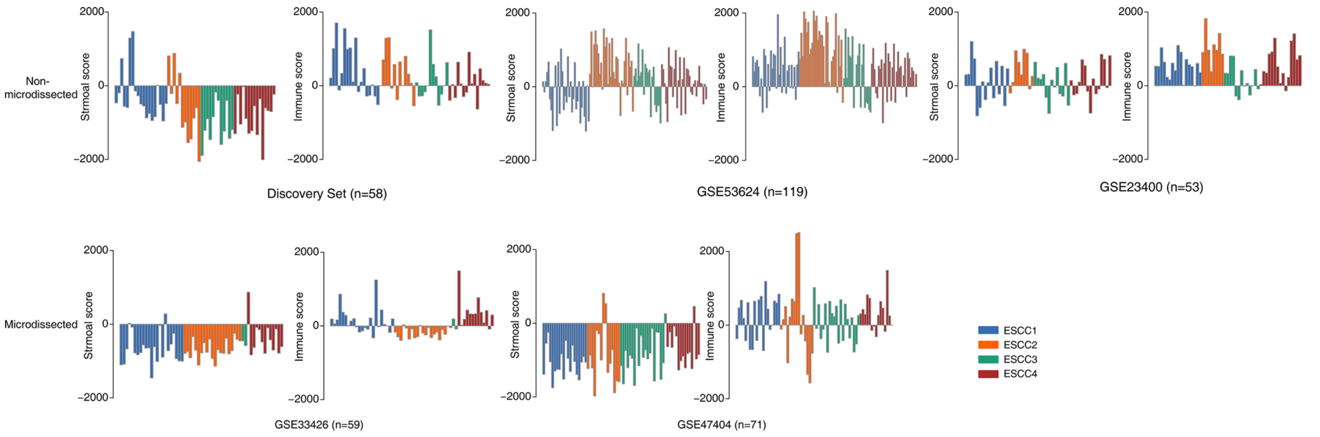

Supplementary Figure 4: Barplots display stromal and immune score for discovery set and validation sets across four subtypes.

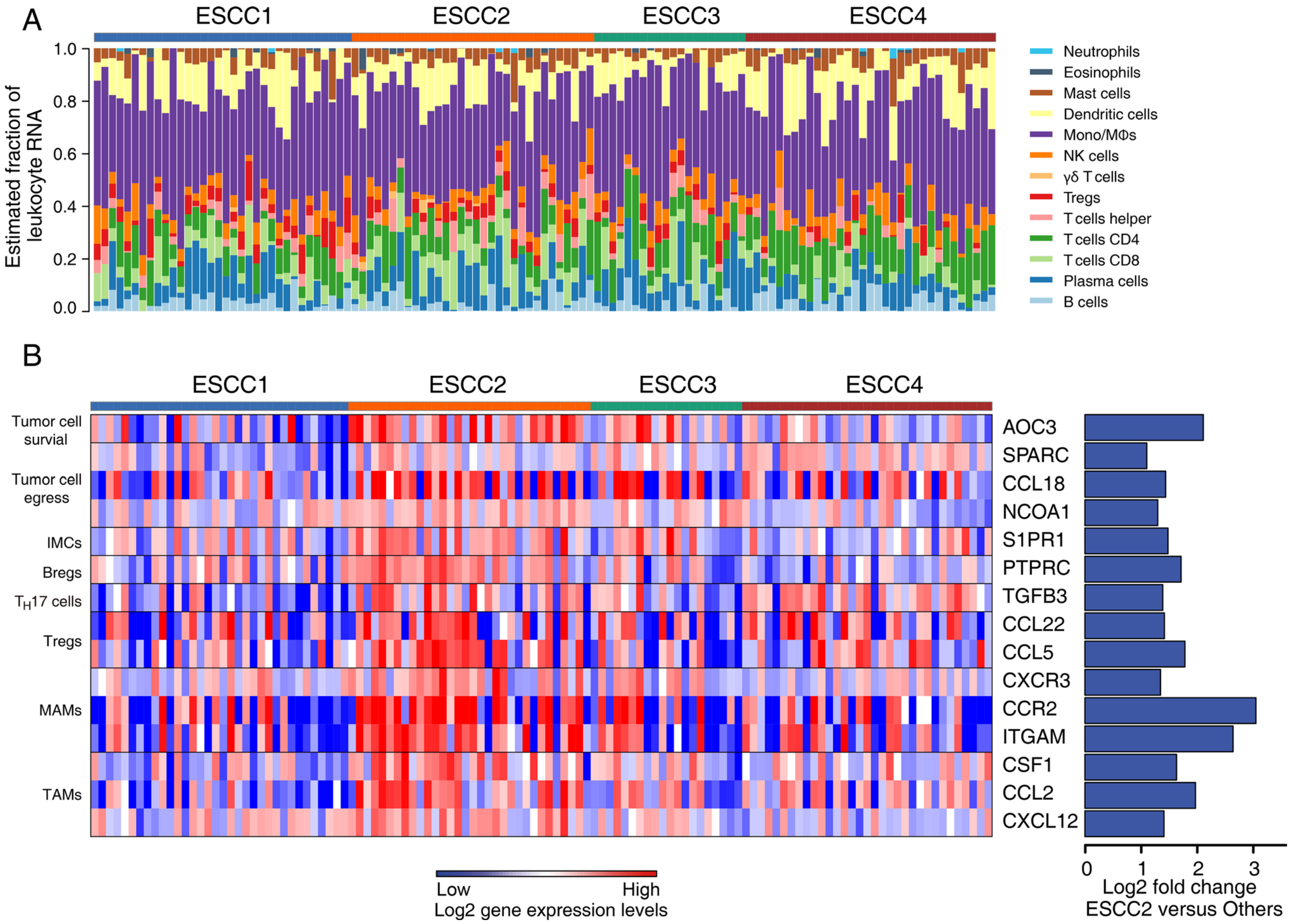

Supplementary Figure 5: Immune analysis for GSE53624. (**A**) Evaluated mRNA fraction of 22 leukocytes across 119 ESCC tumors. (**B**) Heatmap shows the 15 up-regulated genes associated with metastasis in ESCC2.

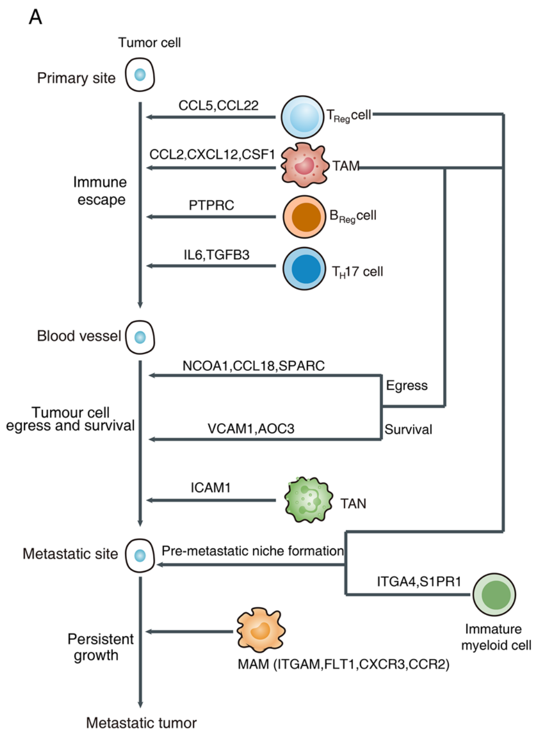

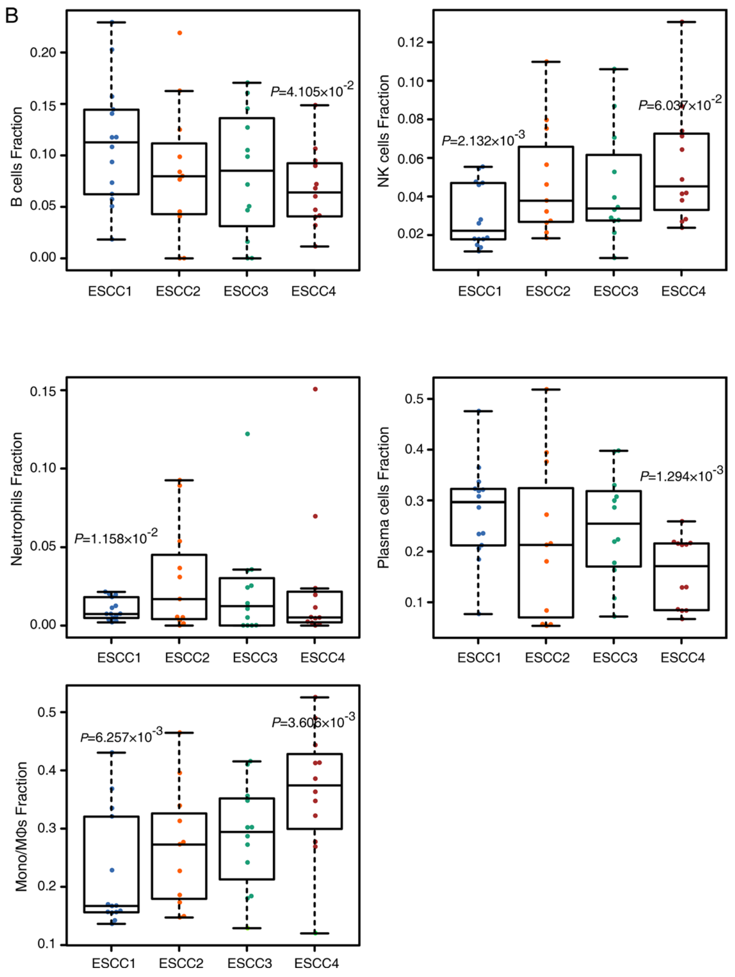

Supplementary Figure 6: (**A**) Immune cells promote each step of metastatic cascade. (**B**) Comparison of immune cell fraction across four ESCC subtypes in discovery set.

Supplementary Table 1: Overview of datasets used in our analysis

Accession number	Samples	Array or Platform	Reference	Treatment	Microdissected
GSE38129	30	Affymetrix Human Genome U133A 2.0 Array	Hu et al., 2015	No	No
GSE45670	28	Affymetrix Human Genome U133A Plus 2.0 Array	Wen et al., 2014	Yes	No
GSE23400	53	Affymetrix Human Genome U133A Array	Su et al., 2011	No	No
GSE33426	59	Affymetrix Human Genome U133A 2.0 Array	Yan et al., 2012	No	Unique Strategy
GSE47404	71	Agilent-014850	Sawada et al., 2015	Unknown	Yes
GSE53624	119	Agilent-038314	Li et al., 2014	No	No

Supplementary Table 2: Signature gene classifier for ESCC subtypes and their PAM score. See Supplementary_Table_2.

Supplementary Table 3I: Clinical information of 58 ESCCs. See Supplementary_Table_3I.
Supplementary Table 3II: Clinical information of GSE53624 (n = 119). See Supplementary_Table_3II.

Supplementary Table 4: Significantly enriched gene sets for ESCC subtypes. See Supplementary_Table_4.

Supplementary Table 5: 89 EMT-core gene list (49 upregulated and 40 downregulated) which expressed in discovery set. See Supplementary_Table_5.

Supplementary Table 6I: CIBERSORT-inferred fractions of tumor-associated leukocytes in discoverset. See Supplementary_Table_6I.

Supplementary Table 6II: CIBERSORT-inferred fractions of tumor-associated leukocytes in GSE53624. See Supplementary_Table_6II.
